# A computational model for predicting changes in infection dynamics due to leakage through N95 respirators

**DOI:** 10.1038/s41598-021-89604-7

**Published:** 2021-05-21

**Authors:** Prasanna Hariharan, Neha Sharma, Suvajyoti Guha, Rupak K. Banerjee, Gavin D’Souza, Matthew R. Myers

**Affiliations:** 1 Division of Applied Mechanics, Office of Science and Engineering Laboratories, Center for Devices and Radiological Health, US Food and Drug Administration, Silver Spring, USA; 2 University of Cincinnati, 2600 Clifton Ave., Cincinnati, OH 45221 USA

**Keywords:** Biomedical engineering, Health care

## Abstract

In the absence of fit-testing, leakage of aerosolized pathogens through the gaps between the face and N95 respirators could compromise the effectiveness of the device and increase the risk of infection for the exposed population. To address this issue, we have developed a model to estimate the increase in risk of infection resulting from aerosols leaking through gaps between the face and N95 respirators. The gaps between anthropometric face-geometry and N95 respirators were scanned using computed tomography. The gap profiles were subsequently input into CFD models. The amount of aerosol leakage was predicted by the CFD simulations. Leakage levels were validated using experimental data obtained using manikins. The computed amounts of aerosol transmitted to the respiratory system, with and without leaks, were then linked to a risk-assessment model to predict the infection risk for a sample population. An influenza outbreak in which 50% of the population deployed respirators was considered for risk assessment. Our results showed that the leakage predicted by the CFD model matched the experimental data within about 13%. Depending upon the fit between the headform and the respirator, the inward leakage for the aerosols ranged between 30 and 95%. In addition, the non-fit-tested respirator lowered the infection rate from 97% (for no protection) to between 42 and 80%, but not to the same level as the fit-tested respirators (12%). The CFD-based leakage model, combined with the risk-assessment model, can be useful in optimizing protection strategies for a given population exposed to a pathogenic aerosol.

## Introduction

In the event of a pandemic or bio-terror attack, personal protective equipment (PPE) such as N95 respirators constitutes an important line of defense against hazardous bio-aerosols. The effectiveness of PPE is strongly dependent upon its ability to prevent aerosol leakage through gaps between the human face and the barrier, in addition to the intrinsic penetration through the respirator’s porous layers^1–3^. The leakage through gaps can be significantly reduced by performing fit testing and selecting an appropriately sized respirator. However, during a public health emergency, fit-testing of respirators may not be possible, and inward leakage of aerosols through the gaps could compromise the effectiveness of the PPE. It is important to quantify the effect of the barrier compromise on the rate of spread of infection.

**Figure 1:**
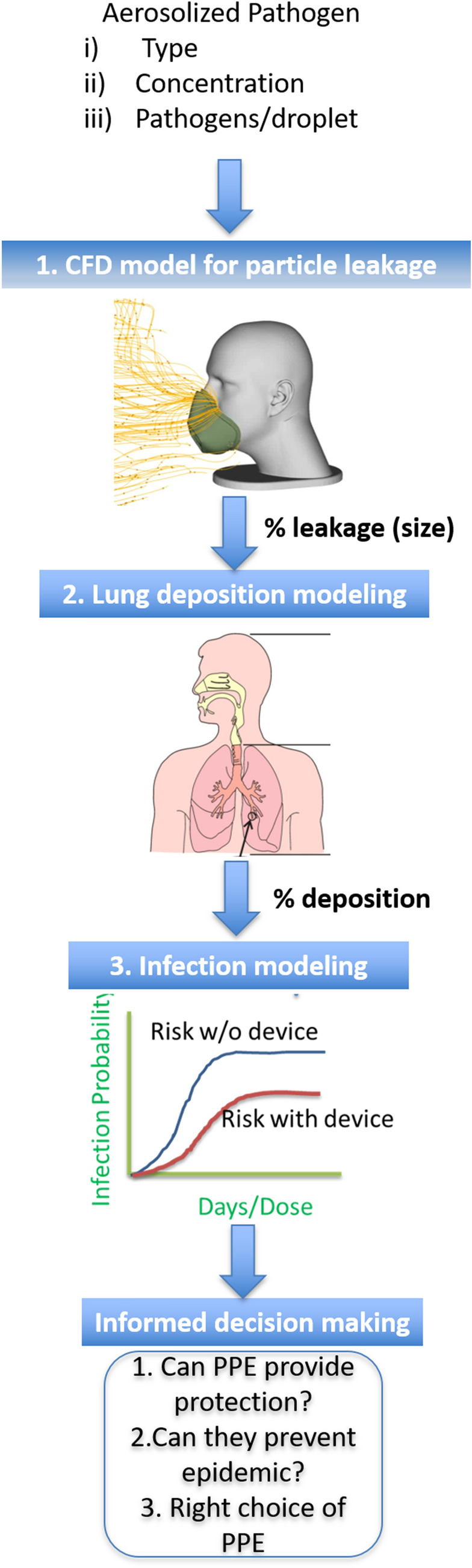
A comprehensive risk assessment model.

We evaluate the increase in infection rate arising from barrier compromise using a strategy that involves three separate models (Fig. 1).

(i) Inward leakages are determined for a variety of facial profiles using a validated computational fluid dynamics (CFD) model,
(ii) Inward leakage values are linked to a one-dimensional lung-deposition model to determine particle deposition at various locations in the lungs
(iii) Deposition results are linked to a risk-assessment model that can predict the infection risk to a population due to the deposited particulates during a pandemic or terrorist attack.

The incorporation of the risk-assessment model helps inform decisions regarding countermeasures to a given threat; one such decision would be the type of respirator to stockpile to protect a given population. The lung deposition model formulated by Guha et al.^4^ is a modified version of the International Committee for Radiological Protection (ICRP) lung deposition model for bio-aerosols. The infection spread model is derived from the SIR (susceptible-infected-removed) model formulated by Myers et al.^5^. The present paper focuses primarily on the development of the leakage-prediction model; details regarding the lung-deposition model and risk-assessment model can be obtained from previous publications^4,5^.

Leakage of aerosols through N95 respirators has been studied by several research groups^1,6–8^. Some experimental and computational studies have examined the leakage of aerosols through gaps created by insufficient sealing between the face and the mask^2,9–16^. Rengasamy et al.^1,2^ studied the leakage of particulates through artificially created leak sites. They measured the total inward leakage (TIL) for particles ranging in size from 8 to 400 nm, for flow rates between 8 L per minute and 40 L per minute (LPM). Leakage between 5 and 20% was measured, depending upon the particle size and the flow rate. Zaripov et al.^9^ used a computational model involving a spherical geometry and reported how TIL increases with increase in the leakage surface area. Oestenstad et al.^10^ used fluorescent tracer particles to identify the location and shape of respirator leaks on half-mask respirators and found that about 89% of the leaks occurred in the nose and chin areas of the face. Coffey et al.^17^ measured the TIL for 21 models of N95 respirators on 25 different subjects and observed that the TIL value could be as high as 88% in the absence of fit testing. Lei et al.^18^ and Cai et al.^15^ developed a “contact pressure” model to estimate gaps between the face and N95 respirators. Cai et al.^14^ evaluated the effect of facial expression on contact characteristics between an N95 filtering facepiece respirator and a headform. Their study showed that contact areas varied with different facial expressions, and facial expressions significantly altered contact pressures and leakage. Lei et al.^12^ extended their contact model and performed CFD simulations to predict aerosol leakage through the gap areas^12^. They also simulated the effects of head movement on the leakage sites between headforms and N95 filtering face piece respirators^13^. They reported that the majority of the leaks happen in the nose and cheek areas.

Most prior studies used a two-step modeling process to predict the leakage of N95 respirators. The first step involved contact pressure modeling (using Finite Element Analysis (FEA)) to simulate the deformation of the respirator on to the headform. The gap geometry between the headform and the respirator was obtained during this step. Subsequently, CFD modeling was performed to simulate the airflow and aerosol leakage through the gaps between the face and the respirator. One consequence of this two-step approach is that errors and uncertainties in the contact modeling are propagated to the CFD modeling. So, the uncertainties in the predicted leakage % can be attributed to uncertainties in model form and input parameters for both the contact FEA and CFD models. This approach precludes separate assessment of the credibility of the FEA and CFD models.

The objectives of the present study were four-fold. First, we developed a new approach for obtaining the gap geometry that does not require contact modeling. Rather than predict gap areas through computational models, we make direct measurements of gap features using mannequins and actual respirators. We performed computed tomography (CT) scans on facial geometries^19^ donned with different commercial N95 respirators. The CT images were then reconstructed to obtain a meshable geometry that contains the gap and the inner dead space volume between the headform and the respirator. Our second objective was to perform CFD simulations on the reconstructed geometry to determine how the leakage is affected by the various fitting deficiencies that were measured.

The third objective was to validate the image-based modeling approach through two different experiments:

(i) Initially, validation experiments were performed by attaching the respirators with circular holes to flat plates. The flat plate mimics the headform and the circular holes mimic the leakage sites. Aerosol leakage and penetration measurements were made using a water-based condensation particle counter^7^.
(ii) Subsequently, additional experiments were performed by donning different respirators on three different adult headforms^19^. The experimental and computed penetrations were compared. The validation data can be used by CFD modelers performing similar type of simulations.

Our final objective was to translate the computed aerosol leakage to infection risk. For the risk assessment, a specific infection scenario was required. We considered an influenza outbreak in which 50% of the population deployed respirators. However, the approach is quite general and applicable to many other types of infection scenarios, including other pathogens and levels of protection.

## Methodology

### Headforms and respirators

**Figure 2:**
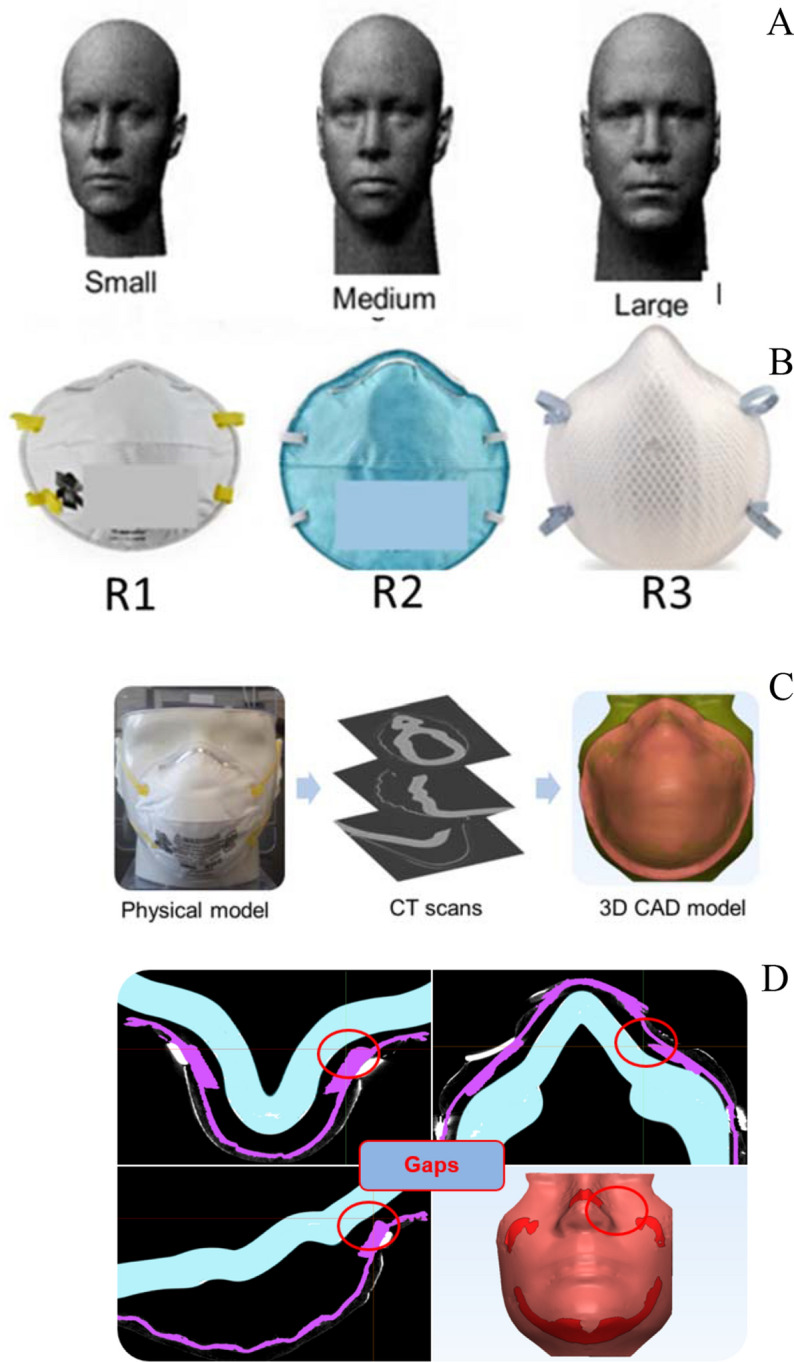
(**A**) Three adult mannequins (**B**) Three mask models (**C**) Flowchart showing the image-based procedure (**D**) sample gap between the face and the respirator.

Headforms for our leakage study (Fig. 2a) were obtained from the National Institute for Occupational Safety and Health (NIOSH), which developed a panel of headforms for fit testing using the principal component analysis method^19^. This panel classified subjects into five categories of head size: small, short/wide, medium, long/narrow, and large. The geometric data of the headforms were obtained from an antropometric survey of 3,997 US workers, conducted at the National Personal Protective Technology Laboratory at the Center for Disease Control. Three of the five headforms from the NIOSH project were used in this study. Physical models of the three headforms were built in Acrylonitrile Butadiene Styrene (ABS) plastic using the Fused Deposition Modeling (FDM) technique^20^, which is a commonly used additive manufacturing technology for printing of parts and devices. The additive manufacturing was performed at Materialise Inc. (Ann Arbor, MI). The minimum dimensional tolerance for the FDM technique was estimated to be 0.1 mm. The surfaces of the headforms were also smoothed to help ensure that the roughness of the surface didn’t impact the CT scanning process or the leakage-measurement experiments.

Three respirator models (Fig. 2b), labeled R1, R2, and R3, were chosen for this study. The respirators were produced by two manufacturers. The respirators were all N95 filtering facepiece respirator type, as certified by the National Institute for Occupational Safety and Health (NIOSH). These respirators typically have multiple size options (small, medium, large). We choose respirator models with different surface areas to produce different gap profiles, and consequently different levels of protection, when combined with the different facial types used in our study. The surface area of the three respirators exposed to particles was estimated to be 156 cm^2^, 192 cm^2^, and 213 cm^2^ for R1, R2, and R3, respectively. The areas were measured by drawing horizontal and vertical lines spaced 1 cm apart throughout the outer surface of each respirator, and then counting the number of squares that were created from the intersection of these lines. As the study was not intended to be a respirator comparison, only the single size of each type was included.

### Computational model

#### CT scanning to capture the leakage gaps

The physical models, comprised of the respirator fitted to the headform, were converted to 3D computer-aided design (CAD) models using an image-based methodology outlined in Fig. 2c^21,22^. Computed tomography (CT) scans (North Star Imaging, Inc.) were performed on the headforms after the respirators were attached. The inner surfaces of the masks were sputter coated with gold (one-micron thickness) to ensure that the mask surfaces were discernible in the CT scans. The spatial resolution of the CT scans was 75 µm. A total of nine scans were performed to cover all mask-headform combinations. Figure 2d shows a sample scan that outlines the mask and gaps near the cheek region of the face. For one headform and mask, the scans were repeated three times after removing and re-donning the mask. This was done to determine whether the leakage area and the % leakage were sensitive to variabilities in the donning process.

#### Conversion of CT scans to 3D computer-aided design (CAD) geometry

After CT scanning, image reconstruction was performed to convert the stacked CT slices to a 3D CAD geometry for CFD simulations (Mimics and 3-matic, Materialise, Inc.). The reconstruction process involved the following steps:

(i) *Thresholding* – From the CT images, the face, respirator, and the air-gap between them were tagged separately based on the variations in pixel intensities (in Hounsfield units).
(ii) *Region growing* – The tagged pixels belonging to each of the three regions of interest (face, respirator, and the air-gap) were separated using a ‘region growing’ operation. Thresholding and region growing complete the segmentation portion of the image processing.
(iii) *Reconstruction* – Following segmentation, the 2D image was reconstructed into a 3D stereolithographic (STL) volume representing the desired geometry.
(iv) *Post-processing* – The geometry in the STL file was subjected to additional wrapping and smoothing operations while ensuring that the gaps and contact points between the face and respirator were unaffected during post-processing. An image of the reconstructed and post-processed 3D CAD model is shown in Fig. 2c.

Subsequently, total gap surface area normal to the airflow direction was quantified using the 3-matic software (Materialise, Inc.)^21^.

#### Obtaining the CFD geometry and mesh

**Figure 3:**
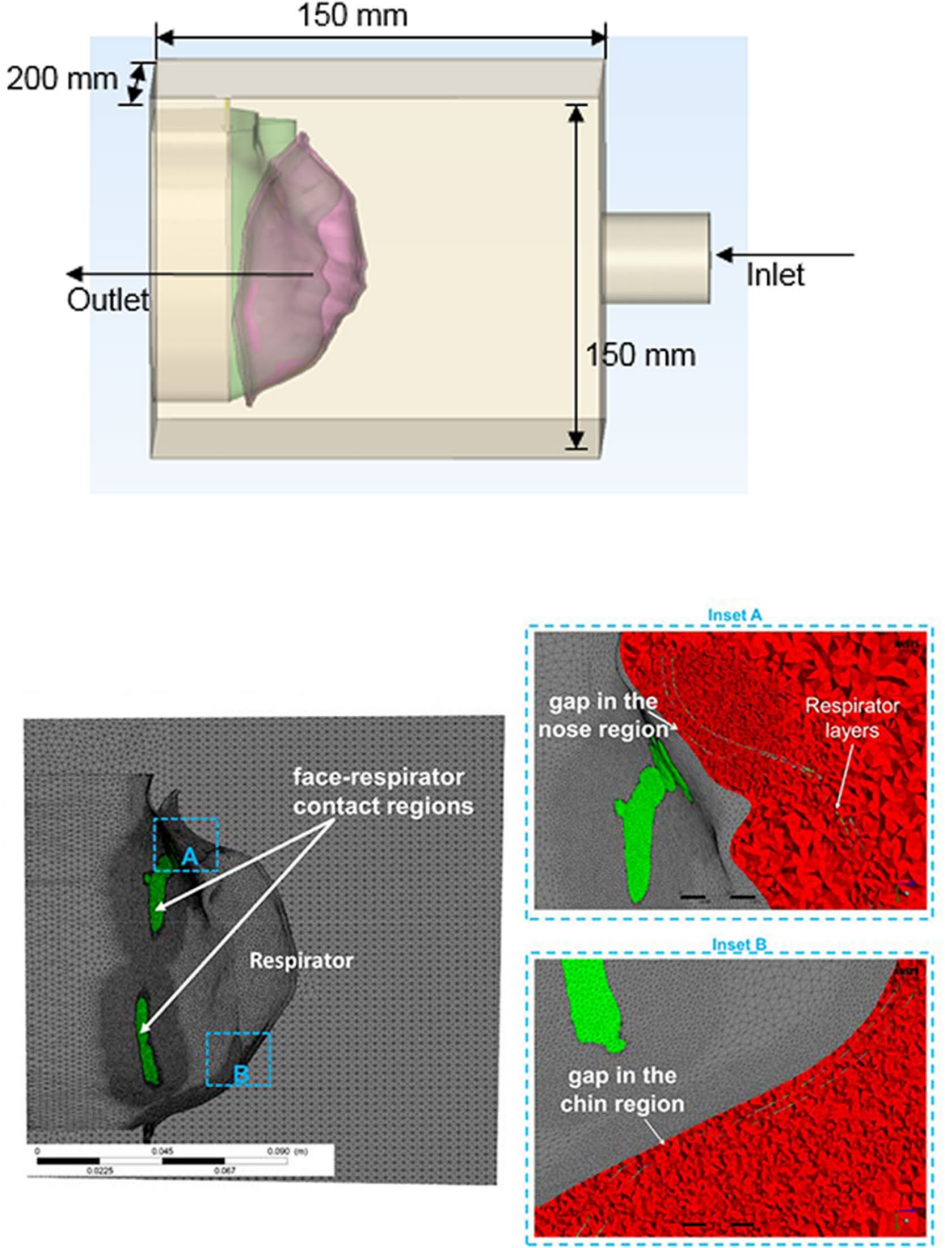
(**A**) CFD geometry of headform and respirator inside the chamber, (**B**) CFD mesh (sectioned) with insets (**A**) and (**B**) showing the gaps in the nose and chin regions.

Figure 3 shows the CFD geometry containing the headform, respirator, and a surrounding enclosure into which aerosols are introduced. In order to render the execution time of simulations manageable, the computational enclosure was reduced in size relative to the enclosure used in the experiments. The dimension of the computational volume was 150 × 150 × 200 mm^3^. Inlet and outlet tube dimensions (10 mm radius) were those of the experimental apparatus. The headform geometries required for the CFD simulations were created using the 3D-reconstructed models described in the previous sections. The fluid domain was meshed into finite volumes consisting of tetrahedral elements (3-matic, Materialise Inc.). The mesh around the face-respirator interface was refined to capture the leakage flow through the gaps. The volumes ranged in size from 1.7 × 10^–8^ m^3^ to 6.8 × 10^–14^ m^3^, depending upon the mesh location (Fig. 3). The total discretized domain contained an average of 20 million tetrahedral elements. The computational geometry was exported for processing to a CFD solver, CFX (Ansys, Inc.). Since the CAD geometry is different for all the CT scans, a unique CFD mesh was created for each face-respirator combination, and the fluid flow and particle transport simulations were performed for all the meshes In addition, the uncertainty arising from inexact knowledge of the gap profile was captured by performing 3 separate trials involving placement of the same mask on the same headform followed by CT scans. The three different gap profiles were meshed again to create additional CFD models. Mesh dependency was checked by increasing the number of elements by 30% over the previous mesh and comparing results. The mesh with the larger number of elements showed less than 1.5% difference in particle-transmission values.

#### Leakage estimation: three-step simulation process

The amount of aerosol transported through the gaps depends on the complexity of the path taken by the aerosol through the gaps, and the relative flow rates through the gaps and the respirator. A 3-step numerical methodology was adopted for determining the porosity, flow split, and aerosol leakage through the face-respirator geometry.

(i) Determination of the porous-medium properties. Airflow through the porous layers of the respirator was modeled in Ansys CFX by introducing a momentum loss term in the governing equation^22^. The porosity and linear resistance coefficient of the respirators, which are the porous-media properties required in the momentum loss term, were obtained in the following manner. The edges of the respirator were completely sealed to a flat plate (instead of a headform) to ensure that all the aerosol penetration occurred through the porous layers of the mask. Subsequently, experiments were performed to measure the pressure drop (magnahelic pressure gauges, models 2006 and 2015; Dwyer Instruments Inc, Michigan City, IN) across the respirator as a function of the inlet flow rate (10 LPM and 70 LPM). The mask porosity and the linear resistance coefficient were adjusted in the CFD simulations until the pressure drop estimated by CFD matched with the experimental data. Following this procedure, the porosity and the linear resistance coefficient values for masks R1, R2 and R3 were determined to be 0.5 and 93,700 kg/(m^3^.s), 0.5 and 89,800 kg/(m^3^.s), and 0.5 and 2,400,000 kg/(m^3^.s), respectively. While the resulting material porosity may not match measured values, an exact match was not required for the limited purpose of producing the experimental pressure drop.
(ii) Airflow simulation through realistic gaps. In this step, airflow through the gaps between the face and the respirator and through the porous layers of the respirator was simulated by solving mass and momentum conservation equations. The CFD meshes for the simulations were obtained from the reconstruction process described in the previous sections. The respirator was modeled as a porous medium with the penetration properties obtained from step#1. Consistent with the experiments, breathing flow rates of 10 LPM and 70 LPM were used in the simulations. Inhalation is assumed to happen through the mouth. These values represent adult respiration rates at rest and during exercise^4,7,23^. For 10 LPM, the air flow was assumed laminar and simulated using the Navier–Stokes equations. For 70 LPM, the air flow was assumed turbulent and a SST k-$\omega$ model was used for the simulation^24^. The k-$\omega$ model is designed to treat low-Reynolds number turbulence, such as that encountered in mask flows. We also chose the SST k-$\omega$ model based on its performance in large-scale round-robin studies on benchmark flow geometries^25,26^. The breathing rate (10 LPM or 70 LPM) was prescribed as an outlet boundary condition. A zero-pressure inlet boundary condition was prescribed. A condition of zero velocity was imposed on the outer boundaries of the computational domain, consistent with the presence of the tank walls in the experiments. With these boundary conditions imposed, the solver computed the airflow through the gaps and the porous layers of the mask.
(iii) Aerosol transport through the leakage sites. The flow field through the leakage sites near the respirators was input to a Lagrangian particle transport model^27^ to simulate the transport of aerosols. Since the airflow through the pores in the respirator layers are not directly modeled, particle transport through the porous media itself was not modeled. Details about the Lagrangian model used in this study are provided elsewhere^22^ but, briefly, tracking was performed out by solving an ordinary differential equation prescribing the trajectory of each particle. The differential equation accounts for the drag force acting on the particle by the air, as well as the gravitational force. Based on the interaction between the discrete phase and the continuous phase, one-way (the air flow influences the particle motion, but not vice-versa) coupling is an acceptable approximation for this study. The value of the coefficient of restitution was set to zero at the walls, based on the assumption that the submicron particles stuck to the wall upon collision. The input parameters for aerosol transport, including the density (2250 kg/m^3^) and aerosol size (100 nm), were obtained from the experimental studies^23^. The aerosol flux was modeled as spherical particles injected from the inlet of the enclosure. A uniform spatial distribution of aerosol particles was assumed at the injection site. After counting the total number of particles exiting the mouth, the % leakage for a specific headform-respirator combination was obtained using the following expressions
  1 $$Leakage \left( \% \right) = \frac{{\mathop \sum \nolimits_{i = 1}^{NP} N_{out, i} }}{{\mathop \sum \nolimits_{1}^{NP} N_{in,i} }}*100$$
  2 $$N_{out,i} = \left( {RT_{max} - RT_{i} } \right) \times I$$
  3 $$N_{in,i} = I \times RT_{max}$$

Here NP is the total number of pathlines used for tracking the particles. RT_max_ is the largest residence time for all the pathlines and I is the particle injection rate. The injection rate can be obtained from the inlet particle concentration and the airflow rate. This expression ensured that the leakage % was estimated after taking into account the differences in residence time of various particle pathlines. The total number of particles entering the domain (i.e. NP) was increased until the leakage % became independent of the particle count.

##### Particle deposition model

A modified version of the International Committee for Radiological Protection (ICRP) lung deposition model was written in MATLAB^4^. This model assumes a monodispersed distribution and considers the impact of shape of a bio-aerosol on lung deposition. In addition, it also accounts for differences in deposition across various age groups. It uses the same empirical equations used for lung deposition modeling in ICRP but derived the correlation between age, height and lung parameters by using clinical data. The model is not able to consider hygroscopicity, and mucociliary clearance rates in the lungs.

##### Risk assessment model

The SIR (susceptible-infected-removed) model developed by Myers et al.^5^ was used to estimate the effect on the infection rate of various levels of respirator leakage. The model outputs the number of infections as a function of time for a population of interest, given the characteristics of the population (size, interaction time, recovery rate, adoption rate for protective equipment…), the pathogen (dimension, settling rate, inactivation rate…) and the protective equipment (inward particle flux, outward particle flux). For the present risk assessment, influenza virus was chosen as the pathogen of interest. All of the population and pathogen characteristics for the simulations were obtained from a prior publication^28^. Yan et al. provided both the source and receiver protection factor of the protective barrier^23^. For this study, the source protection factor of 7 was taken to be that used by Yan et al., while the receive protection factor was estimated from our CFD model as 1/Leakage_Fraction^5,28^. In terms of risk assessment, the purpose of the procedures described in the previous sections is to generate the leakage fraction. The condition used to initiate the infection dynamics is one person out of the population infected with influenza. The number of pathogens per droplet was assumed to be 1.9 × 10^–3^. The compliance rate for using the respirators was assumed to be 50% of the population. The risk assessment model also assumes that the leakage is independent of the particle size.

### Validation experiments

**Figure 4:**
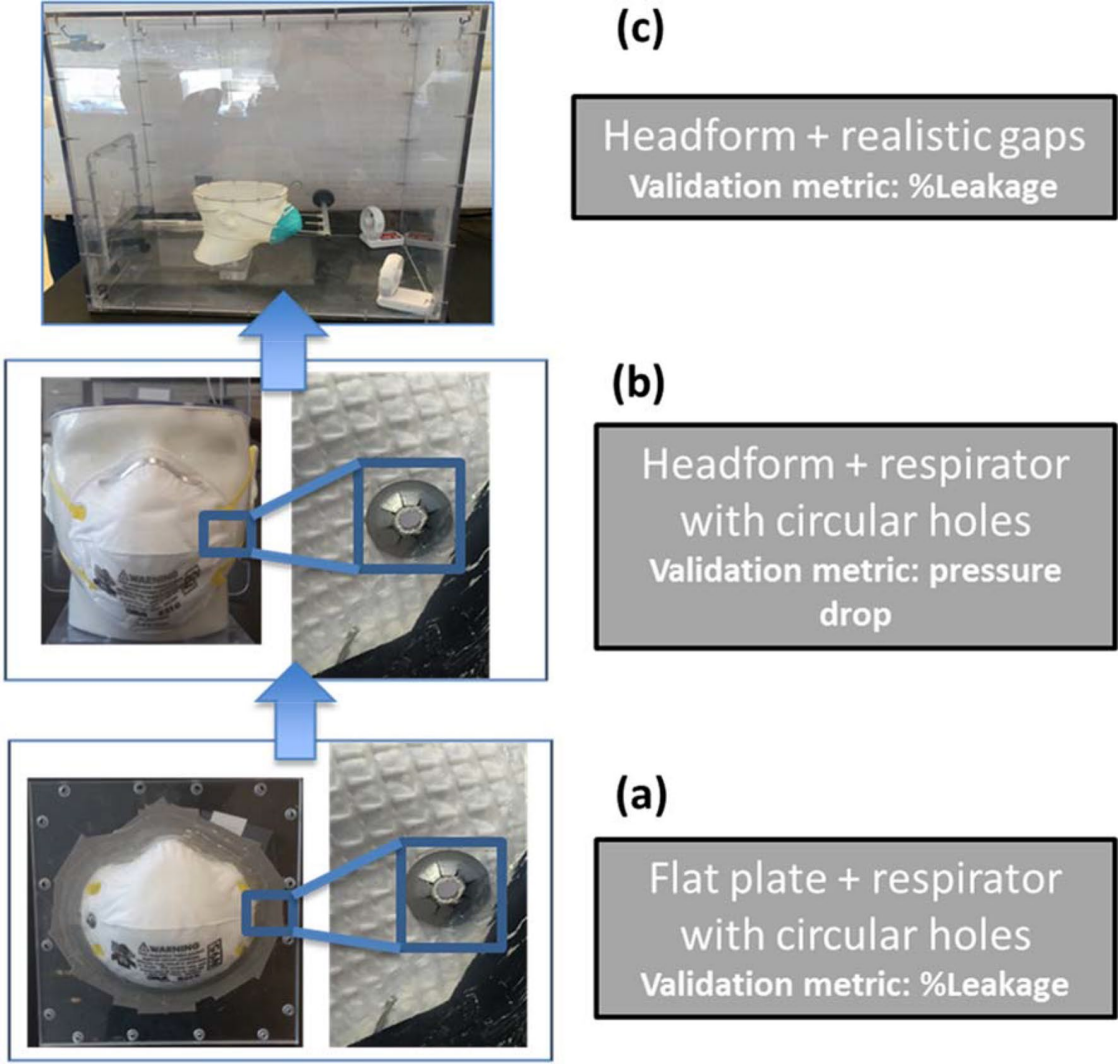
Validation geometries.

The validation of the CFD model was performed in three stages (Fig. 4). First, using a simplified gap of known size, and subsequently using a more realistic gap profile interfacing the headform and the respirator. Details of both the validation studies are provided below.

(i) *Flat plate experiments:* The experiment involved creation of gaps of known shape and dimension at select locations on the respirator*.* The respirator was glued to a flat plate and sealed to avoid any leakage occurring at the interface of the flat plate and the respirator. Subsequently, multiple circular holes (3 mm diameter) were created using a piercing tool (Model:8025-N95, TSI Incorporated, Minnesota) in select locations (Fig. 4). Two probes, one placed upstream of the respirator and one placed downstream, were used to measure the concentration of the aerosols. Further details about this experimental set-up and protocol are published elsewhere^4,7^. The approach for estimating the filtration efficiency and leakage followed the method described in previous publications^7,23^. Briefly, a ~ 1.5% NaCl solution prepared was aerosolized (using a 6-jet collison nebulizer), dried, and neutralized before being sent to the chamber housing the flat plate or headforms to which the N95 respirators were attached. A Scanning Mobility Particle Sizer (SMPS, model#3936, TSI Inc. Minnesota) was used to monitor the concentration of the aerosols upstream and downstream of the respirators. The size distribution of the aerosols ranged from approximately 20 nm to 700 nm. The geometric mean typically ranges from 75 – 90 nm. The penetration and the leakage values were calculated by averaging across the entire size range. Detailed calculations are provided in a previous publication^23^.
(ii) *Headform experiments:* The same headforms that were 3D-printed for CT scans were used for the validation experiments. The face/respirator models were placed into a box of dimensions 30 cm × 33.5 cm × 47.5 cm. An inlet tube of radius 10 mm and length 50 mm was attached to the front side of the enclosure (opposite the face and respirator). The outlet was a circular tube of radius 10 mm. Polydispersed NaCl (1.5% concentration) was used for generation of aerosols. Our previous study reported that the size of the aerosols ranged between 18 and 950 nm. A 6-jet collison nebulizer (BGI, Butler, NJ) was used to aerosolize the NaCl solution. A diffusion dryer and a neutralizer were used for drying and charge neutralizing the aerosols. The aerosol was mixed with dried and filtered dilution air and sent to the chamber. This dilution air was controlled using a mass-flow controller (model 65,524; Alicat Instruments, Tuscon, AZ). Two probes, one placed upstream of the PPE and one placed downstream, measured the concentration of the aerosols. The SMPS was employed to measure the aerosol concentration.

In one validation procedure involving headforms, experiments were performed by gluing the respirator to the headform and creating circular-shaped holes of 3 mm diameter to serve as gaps (Figure 4b). The holes sizes were based on previous publications^23^ and were located few centimeters from the outer circumference. The objective of the procedure was to use these experiments as an intermediate validation step to evaluate the accuracy of the CFD simulations. The pressure drop across the respirator (with circular holes) was measured and compared with predictions from the simulations. As the final validation exercise, the percent leakage for the realistic gaps between the headforms and respirator was measured and compared with CFD results (Figure 4a).

For the particle sizes considered in this study, the intrinsic penetration through the respirator fiber was observed to be less than 0.1%^4,7^ and was not considered while estimating the leakage for the simulations.

## Results

### Gap surface area

**Figure 5:**
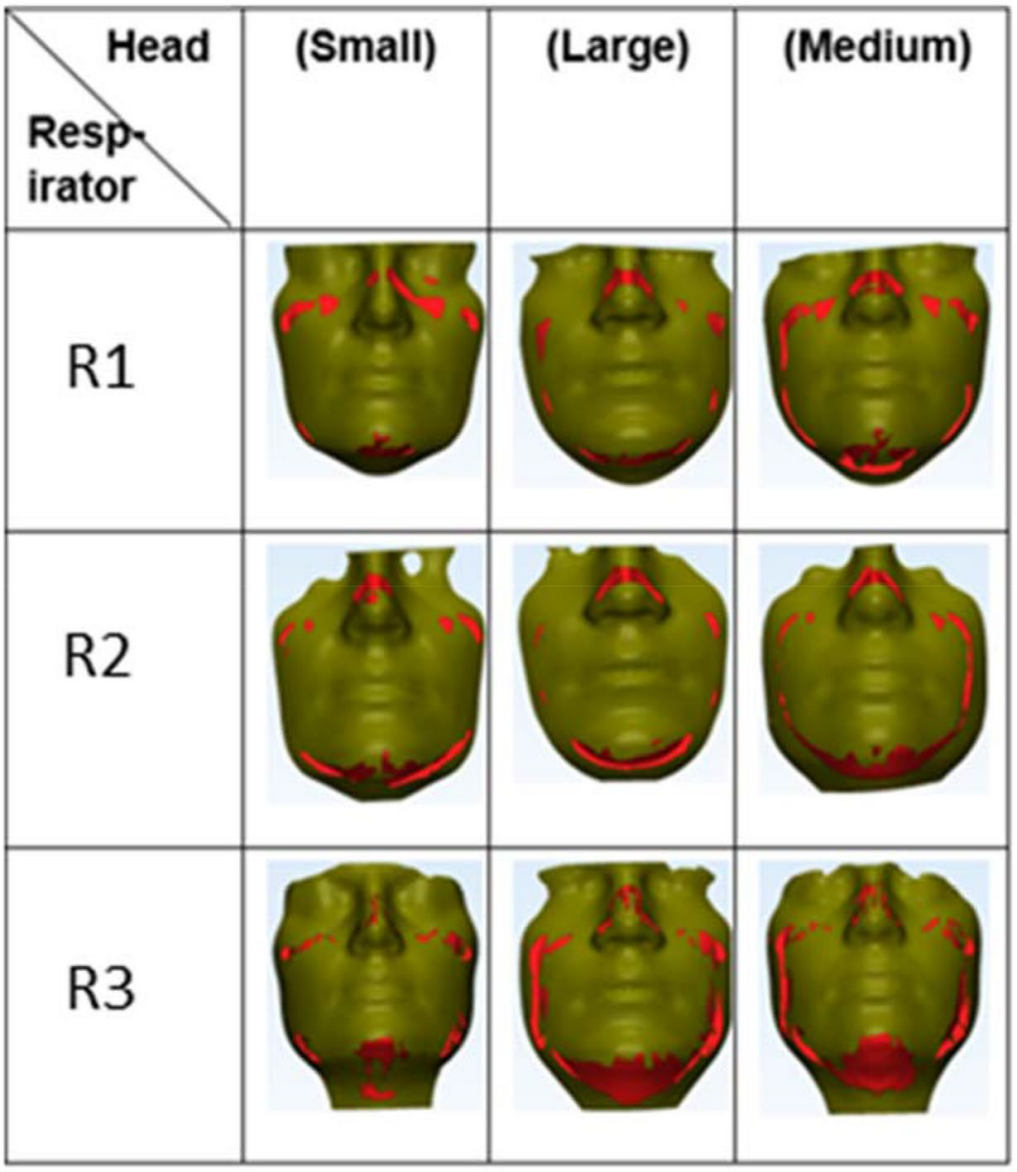
Anatomical leakage sites. Red zones are regions of contact between face and the respirator. The leakage sites are the gaps between the red zones.

Figure 5 shows the leakage sites for all respirator-headform combinations. For all the combinations, there were no gaps at the nose, chin, and the left and right cheek bones. Notably, the gaps were located asymmetrically on the face.

**Figure 6:**
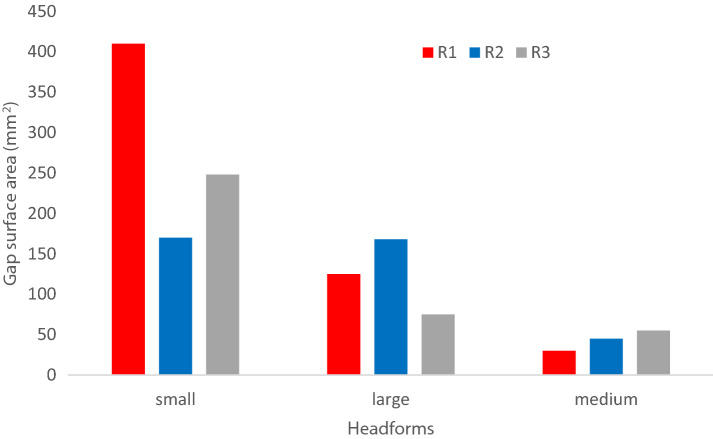
A plot showing the variation of gap surface area with different head-respirator combinations.

Figure 6 provides the gap surface area for the nine head-respirator combinations. The gap surface area varied between 27 mm^2^ and 409 mm^2^. The Medium head was observed to have maximum contact with respirators R1and R2 (gap surface area = 30 mm^2^ and 45 mm^2^, respectively). In contrast, the Small head was observed to have minimum contact with all the respirators and consequently had the highest gap surface area (172 mm^2^ to 409 mm^2^).

### Validation: flat plate-respirator geometry

Table 1 compares the pressure drop across the respirators attached to the flat plate, with each respirator containing the 2 circular holes. On average, the numerical pressure-drop values matched the experimental data within 15%. For all conditions, the CFD models overestimated the pressure drop compared to experiments.

**Table 1:** Comparison of pressure drop across the flat plate-respirator.

Respirator	Q = 10 LPM		Q = 70 LPM	
	Experiment (mm H_2_O)	Computation (mm H_2_O)	Experiment (mm H_2_O)	Computation (mm H_2_O)
R1	0.7 ± 0.06	0.75	5.5 ± 0.25	6.31
R2	0.85 ± 0.1	0.98	7.31 ± 0.6	8.24
R3	0.95 ± 0.3	1.18	9.44 ± 0.3	9.8

**Figure 7:**
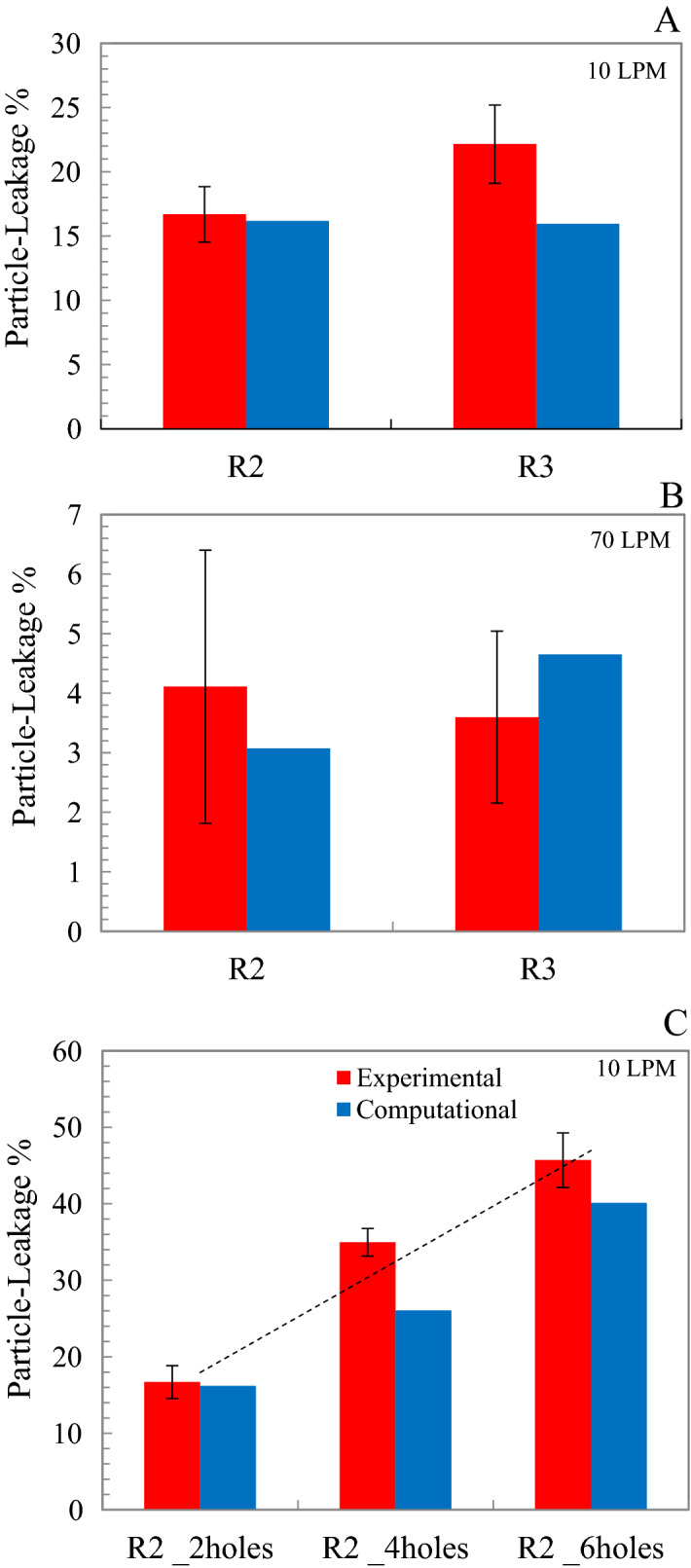
Experimental and computational particle-transmission % for flat plate (**A**) Resting breathing rate of 10 LPM (**B**) Exercising breathing rate of 70 LPM (**C**) For R2 with increase in number of holes at breathing rate of 10 LPM (R^2^ = 0.98); n = 3 for experiments.

Figure 7 shows the experimental and computational aerosol leakage for the flat plate and R2 and R3 respirators, with varying numbers of holes. On average, the leakage values determined by experiments and simulations were within 20% of each other. The minimum difference between experimental and numerical leakage values for a resting breathing rate of 10 LPM (Fig. 7A) was 3%. The maximum was 28%. At a breathing rate of 70 LPM (Fig. 7B), the minimum and maximum discrepancies were 25% and 29%. The amount of leakage was similar for the two respirators (about 15% for 10 LPM and 4% for 70 LPM). As the number of holes in R2 increased, the increase in leakage was approximately linear (Fig. 7C). In general, the CFD model under-predicted the transmission compared to the experiments.

**Figure 8:**
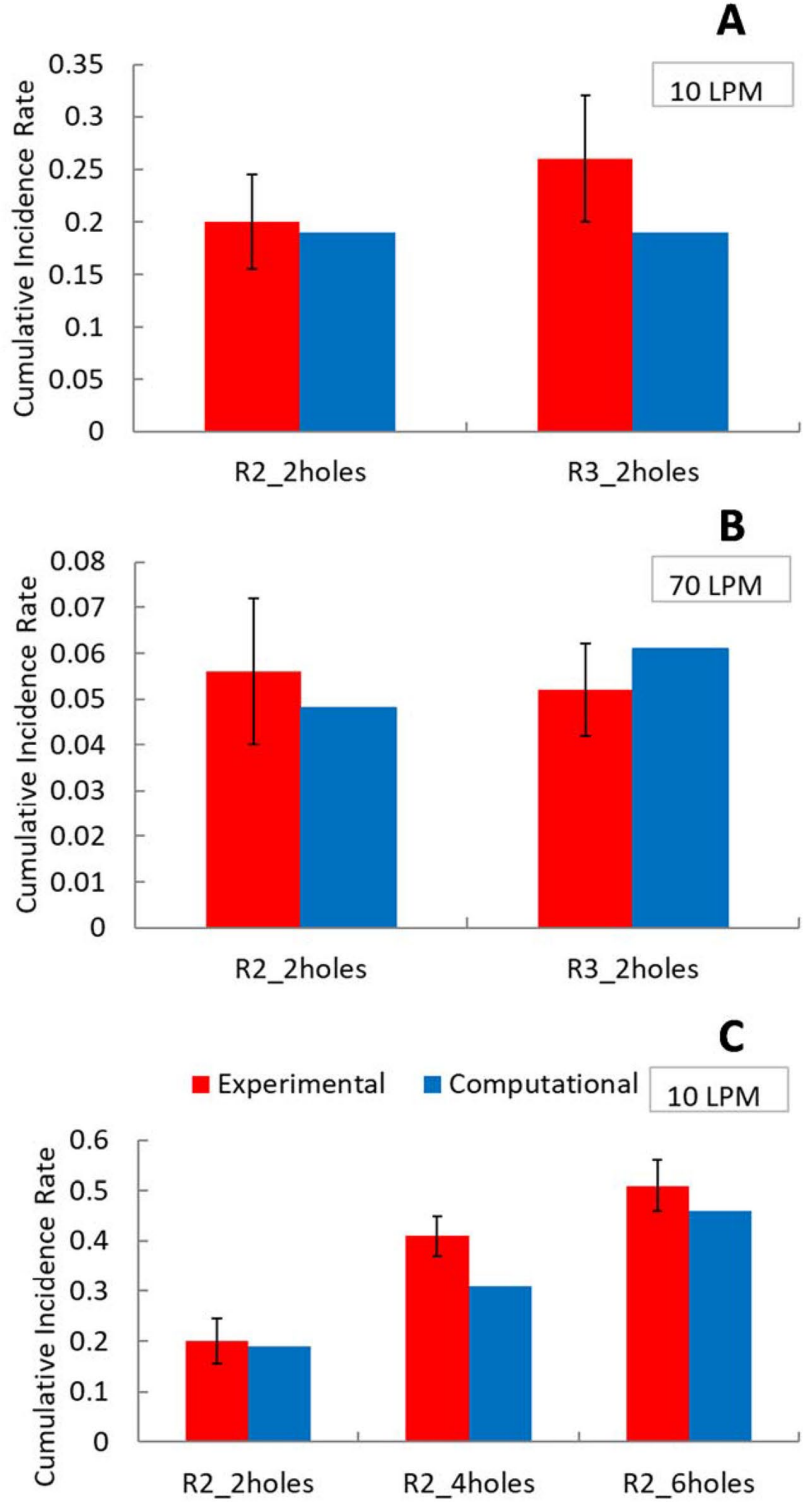
Cumulative incidence rate for experiments and simulations for flat plate with R2 and R3 respirators for breathing rate of 10 LPM; n = 3 for experiments.

Regarding the effect of leakage on infection rate, Fig. 8 shows the cumulative incidence rate (CIR) calculated by the risk-assessment model. The CIR provides the total fraction of population infected by the pathogen. The predicted infection rates were not highly sensitive to the differences in the transmission rate between experiments and simulations. On an average, the CIR values based upon experiments and simulations differed by about 16%. The maximum difference in CIR between experiments and simulations was observed for the R3 respirator (0.26 vs 0.19) at 10 LPM.

### Validation: headform-respirator geometries

**Figure 9:**
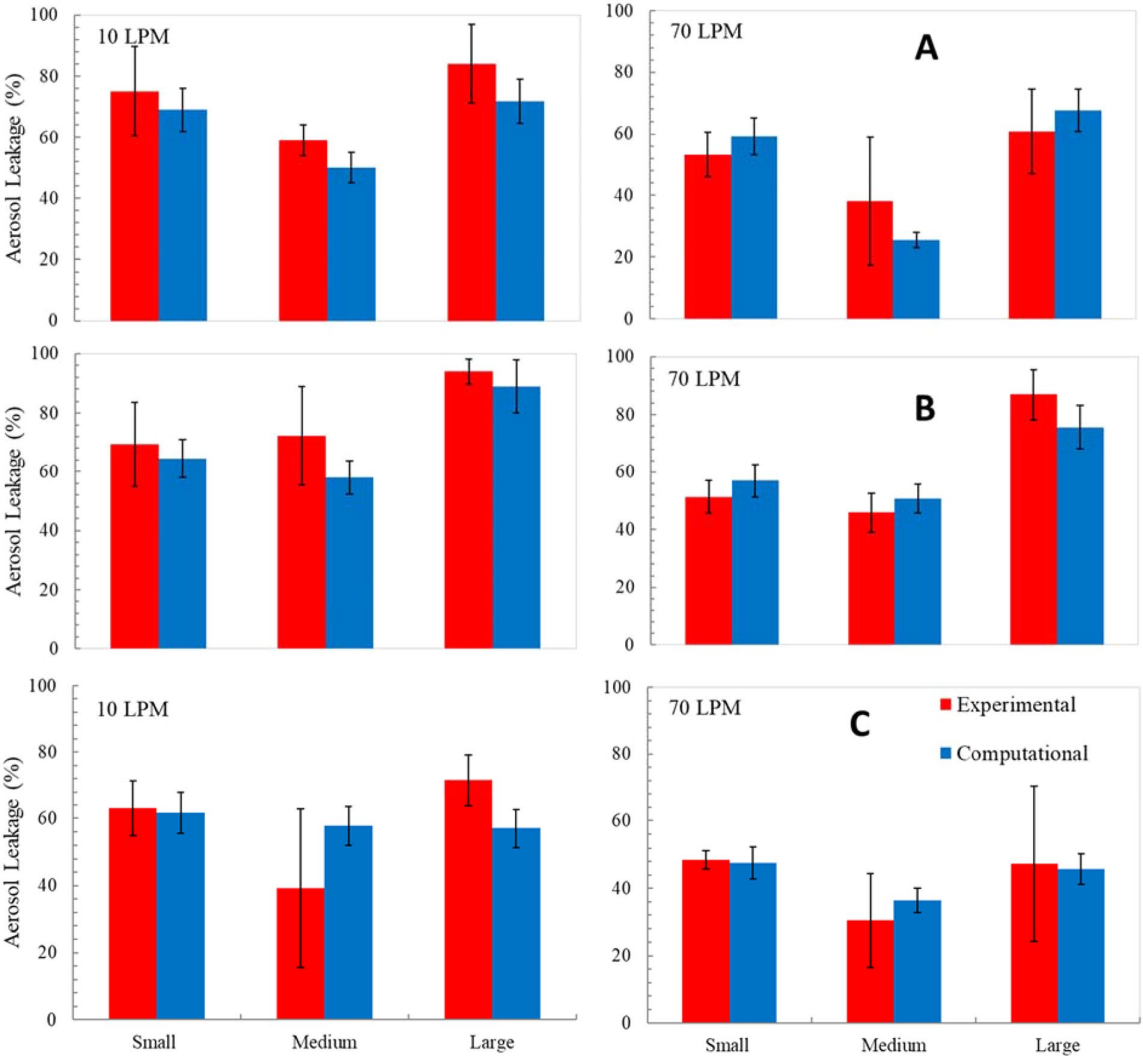
Experimental and computational aerosol leakage percentage for 10 LPM and 70 LPM (**A**) Respirator, R1 (**B**) Respirator, R2 (**C**) Respirator, R3; n = 3 for experiments.

Figure 9 shows bar graphs of the experimental and computational aerosol leakage percentage for the small, medium, and large headforms. For the simulations, the uncertainty arising from inexact knowledge of the gap profile was included, in the following manner. The gap profile was first measured by the CT technique for 3 separate trials involving placement of the same mask on the same headform. The three different gap profiles were input into the CFD model and the variation in leakage was computed. The resulting relative difference in leakage was found to be approximately 10%. This 10% uncertainty was included in the numerical results in Fig. 9. The uncertainty was also incorporated into the input to the lung-deposition and risk-assessment models, to determine an uncertainty in the CIR for the different scenarios considered.

Across all headforms and both flow rates, the average difference in leakage value between the CFD and mean experimental data for R1, R2, and R3 respirators was calculated to be 11%, 16%, and 16%, respectively. CFD underestimated the leakage when compared to experiments for 12 out of 18 headform-respirator combinations.

**Figure 10:**
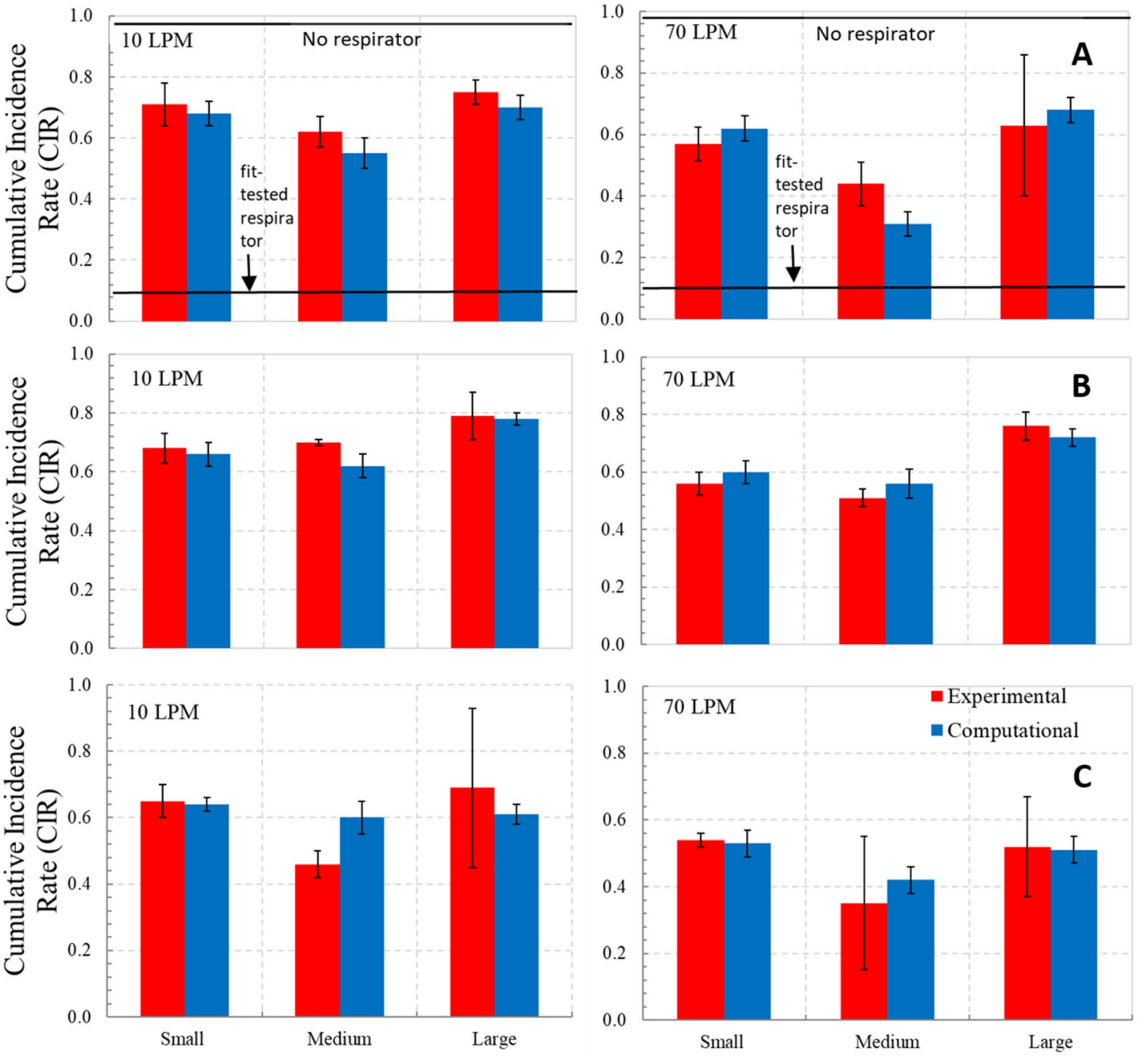
Cumulative Incidence Rates (CIR) from experimental and computational aerosol leakage for 10 LPM and 70 LPM (**A**) Respirator, R1 (**B**) Respirator, R2 (**C**) Respirator, R3; n = 3 for experiments.

Figure 10 shows the corresponding CIR plots for all the headforms and respirators. The average difference in CIR predictions with the risk-assessment model informed by experimental leakage values, compared to when it was informed by computational leakage values, was 7% for R1, 12% for R2, and 13% for R3, though the difference was statistically significant (based upon a two-tail t-test) only for the R2 and R3 respirators with the medium headform at 10 LPM flow rate, and the R1 respirator and medium headform at 70 LPM. For the infection scenario considered in this study, in the absence of protection, 97% of the exposed population was predicted to be infected by the influenza pathogen. In the presence of respirators, depending upon the fit between the headform and the respirator, anywhere between 42 and 80% of the exposed population was predicted to be infected. By comparison, in the presence of a fit-tested respirator, less than 12% of the exposed population was predicted to be infected.

### Effect of gap surface area

**Figure 11:**
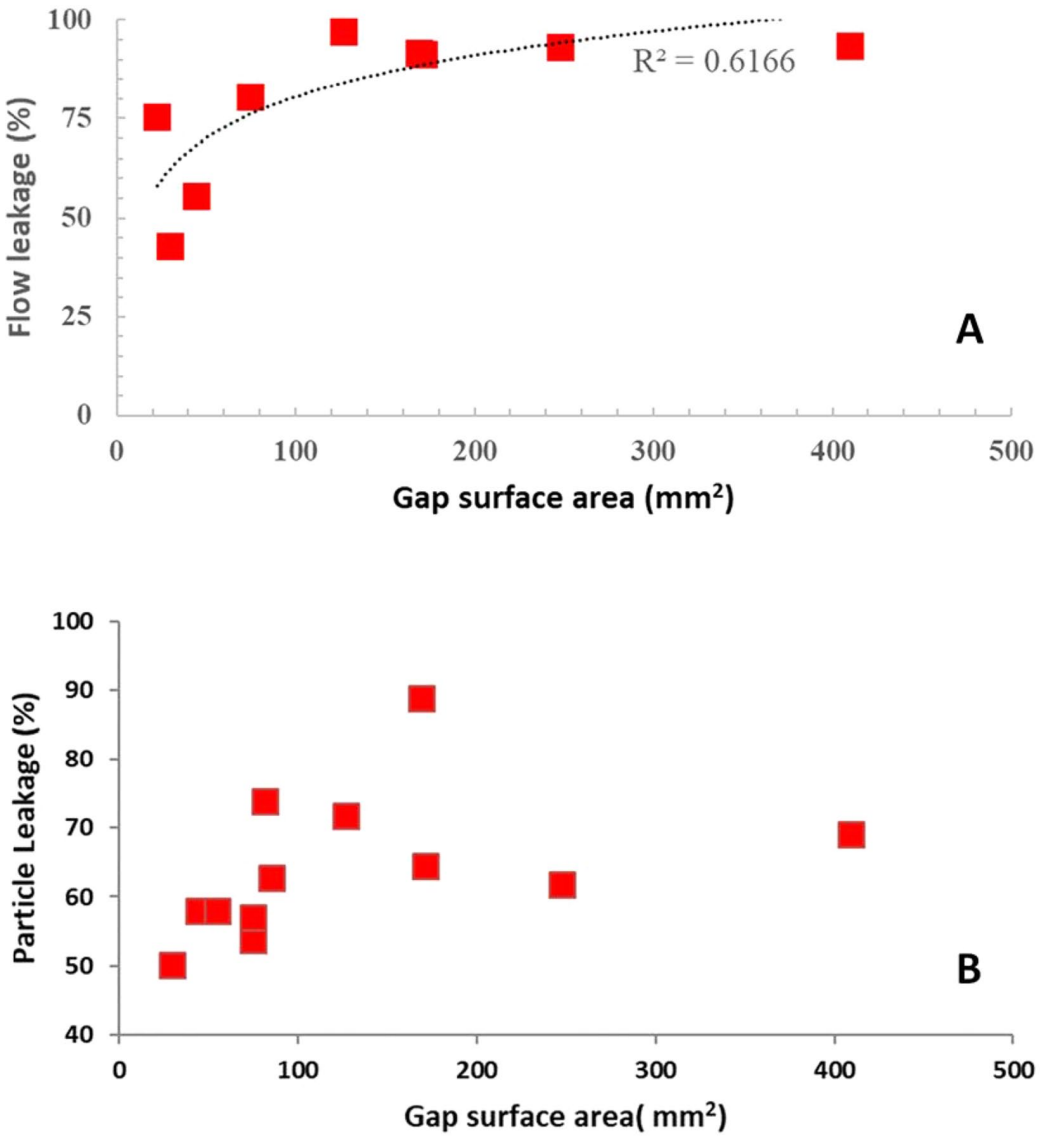
Variation of (**A**) flow and (**B**) particle leakage with gap surface area in mm^2^ for breathing flow rate of 10 LPM (R^2^ = 0.16 for linear fit).

Figure 11A shows variation of flow leakage (%), i.e. the amount of air flowing through the gap, as a function of the gap surface area, for the 10 LPM flow rate. The gap surface area for the nine face-respirator combinations was obtained from image-based modeling and ranged from 0.2% to 3% of the total mask surface area (~ 150 cm^2^). The flow leakage (fraction of flow that is attributed to leakage) corresponding to these gaps varied between 43% and 97% respectively.

**Figure 12:**
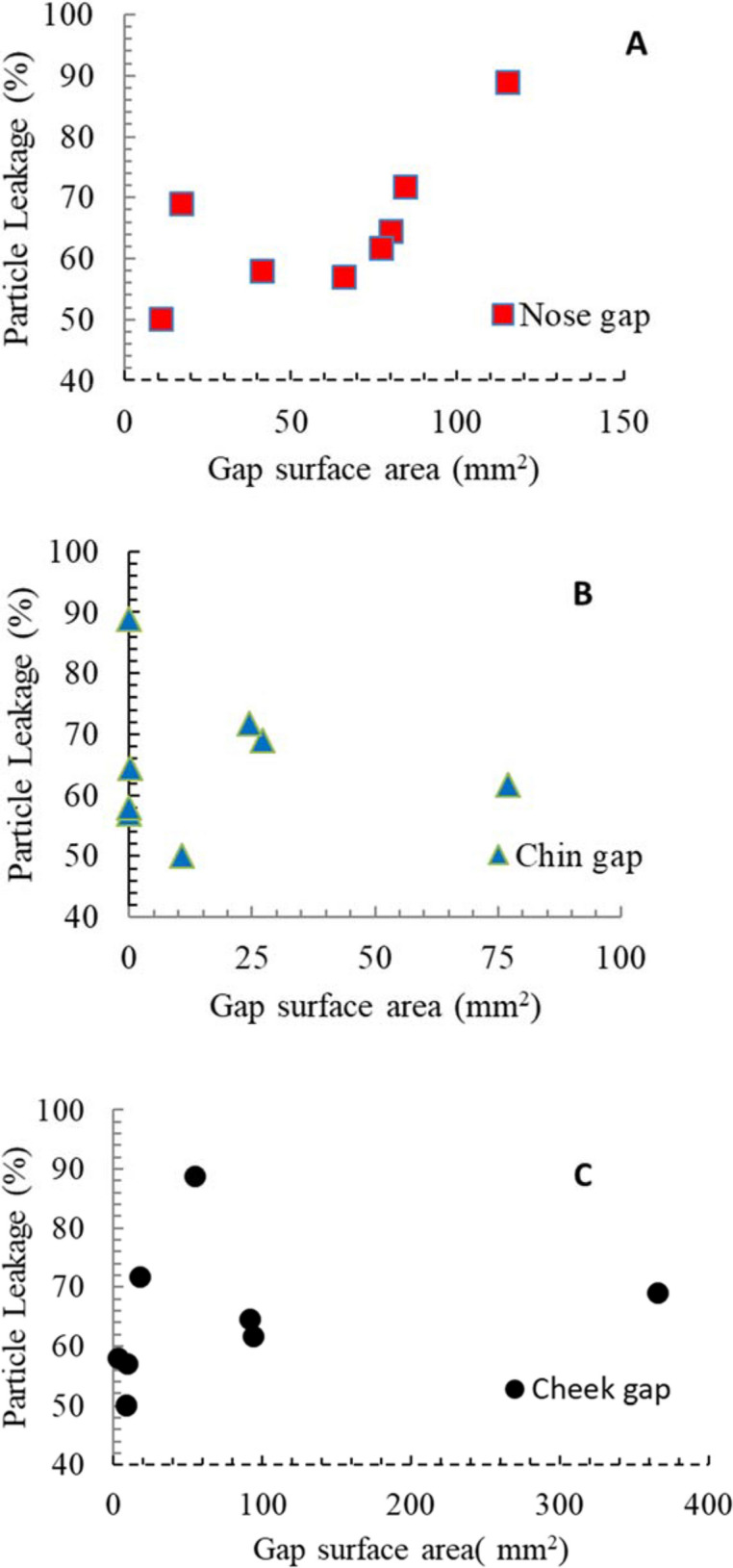
Variation of aerosol leakage as a function of (**A**) nose (R^2^ = 0.48), (**B**) chin (R^2^ = 0.005) and (**C**) cheek (R^2^ = 0.05) gaps.

The correlation of aerosol leakage with gap surface area (Fig. 11b) was weaker than the correlation for flow leakage. When the aerosol leakage was broken up by gap location, a stronger correlation with gap area was observed for the nose (Fig. 12a), and no correlation with gap surface area observed for the chin and cheek (Fig. 12b and c).

### Effect of gaps on spread of infection

To further evaluate the practical implication of the presence of gaps, the SIR model was used to determine the level of compliance required by the population in order to reduce the CIR to less than 5%. With the 50% compliance assumed in the calculations reported earlier, the CIR was estimated to be 0.62, for case of the Medium headform, Respirator R2, and a breathing rate of 10 LPM. Computations showed that 70% compliance would be required in order to reduce the CIR to 5%, about the level of a fit-tested respirator assuming 50% compliance.

## Discussion

This study presented a computational model for evaluating the protection against infection offered by N95 respirators that have not been fit tested. The results from the CFD model, in combination with lung deposition and infection risk models, were used to estimate the risk of infection to the exposed population wearing three different respirator brands.

Our CFD-based leakage protection model was validated against aerosol leakage measurements for various respirator-headform combinations. On an average, the CFD-predicted particle leakage differed from experiments by about 13%. This difference in leakage introduced an uncertainty of 0.05 in the predicted cumulative infection rate (CIR). This uncertainty is an order of magnitude small than the mean infection rate (0.65 ± 0.07) predicted by the model (Fig. 10). In other words, taking in to account the difference between the experiments and the simulations, the approach based entirely upon computations was sufficiently credible to differentiate between the infection risk associated with no protection, a fit-tested respirator, and a non fit-tested respirator.

The variability in gap profile associated with donning technique and respirator/face compatibility was incorporated into the uncertainty associated with the numerical predictions. Other sources of uncertainty were not treated, including the variability in the numerous parameters (subject breathing rate, droplet deposition probability, droplet settling rate… see^23^) contained in the risk-assessment model. Uncertain knowledge of these parameters makes absolute predictions of infection rate difficult. However, for a given population and pathogen, the present model is useful for predicting differences in infection rate with and without fit testing. Regarding propagation of uncertainty owing to variations in gap profile, the calculations revealed that the uncertainty in the infection rate (Fig. 10) was generally less than the 10% uncertainty in leakage rate (Fig. 9) associated with incomplete knowledge of gap profile. That is, when it come to the variability associated with mask fit, risk of infection is less affected than particle transmission.

In order to understand the impact of non-fit-tested respirators, we first consider the ideal scenario of fit- tested respirators. A fit-tested respirator is expected to offer various levels of protection (also referred to as assigned protection factor or APF) that, depending on the application, can range from 10 to 10,000^29^. In context of biological hazards routinely encountered in the workplace, OSHA stipulates that assigned protection factor be 10. In other words, an APF of 10 implies that of the total airborne pathogens in air, an individual donning a fit-tested respirator would only inhale 10% of those aerosols, resulting in a significantly reduced risk of infection compared to the case of no protection. (Note: the APF is not to be confused with fit factor, which is > 100 for a N95 respirator.) From the risk-assessment model, the infection rate when using a fit-tested respirator in the scenario we considered is ~ 12%. Next, for, non-fitted respirators, we need to convert the total inward aerosol leakage values into assigned protection factors. A total inward leakage of 50% corresponds to an APF of 2. For no respirator, the APF is 1. The risk of infection associated with the APFs of 10, 2 and 1 would be 12%, 55% and 97%. The different head-form/respirator combinations studied here provided us with APFs ranging from 4 to 1.05. The corresponding infection rate range from 42 to 80%. These results suggest that non fit-tested respirators offered some level of protection and lowered the infection rate (from 97 to 42%—80%) but not to the same level as the fit-tested respirators. Results also highlight the value of proper fit-testing for N95 respirators, and the risk associated with *en masse* use of non fit-tested respirators during an emergency. Other studies^1,30,31^ have likewise highlighted the increase of risk associated with deploying non-fit-tested respirators.

The CFD-based model is potentially useful for optimizing the level of protection against infection afforded by different protection strategies, for a given population exposed to a hypothesized pathogen. The population characteristics (such as total number of persons, fraction having each facial profile, breathing rate, and inhaled droplet-deposition probability) and pathogen-droplet properties (size, weight, and inactivation rate) would be presumed fixed in the calculations. Variables in the protection strategy that can be controlled, all possessing an associated cost in time or financial resources, include the respirator type, level of fit-testing, and level of compliance. As an example, we consider a population of persons having a large facial profile exposed to an influenza pathogen. Deployment of respirator R2 without fit testing, with 50% compliance, results in a cumulative incidence of infection of 80% of the population (Fig. 10). With Respirator R3, the CIR for the same scenario is 60%. Assuming Respirator R3 is more expensive, public-health officials could use the model to determine the tradeoff between using resources to purchase additional quantities of Respirator R3, or to incentivize the population to increase compliance with Respirator R2 (or some optimal combination of both strategies), in order to reduce the infection rate.

For respirators not having a close fit to the face, aerosol leakage seems to be impacted more by the gaps around the nose area than those near the chin or the cheek gaps. Controlling the nose gap is critical in improving the fit-factor for these respirators. For future respirator designs, or fit-testing procedures, the present model can be used to assess the ultimate payoff (in CIR) for a given gap-reduction effort.

The difference in correlation with gap surface area between flow leakage and aerosol leakage (Figs. 11a and b) was unexpected. Presumably, the large scatter in Fig. 11b for aerosol leakage is due to the fact that aerosol path lines differ from the air streamlines, particularly in the gap regions. In the gap regions, the total gap area is undoubtedly relevant for aerosol transmission. However, the local geometry, including the facial contour at the location of transmission, and the gap height at that location, dictate the amount of deviation from the streamlines, and ultimately the likelihood of adsorption of the particle. The deviation from the air streamlines is presumably most severe in the chin and cheek regions, where there was no correlation with total gap surface area.

The deviation of particles from streamlines is also relevant for evaluating the potential difference in CIR prediction for the 100 nm size NaCl particles used in the study and the 5-micron size influenza droplets assumed in the risk-assessment model. The Stokes number in the gap region provides a measure of the inclination of the particles to deviate from the streamlines, with Stokes numbers greater than 1 indicating the potential for separation. The Stokes number is given by

4 $$\frac{\rho {Vd}_{p}^{2}}{18 \mu D}$$

Here *ρ* is the particle density, *V* the air velocity in the gap region, *d*_*p*_ the particle velocity, *μ* the air viscosity, and *D* the characteristic dimension of the obstacle influencing the flow direction. For both particles, the air velocity in the gap is the flow rate (we initially assume 10 LPM) divided by the gap area (on the order of 100 mm^2^), i.e. about 1.7 m/sec. The viscosity of air is approximately 1.8 × 10^–5^ (N s/m^2^). The characteristic dimension was taken to be the radius of curvature of the tip of the respirator surface, given that the acceleration of the flow around that surface into the gap strongly influences the likelihood that the particle will separate from the streamline. The radius of curvature is comparable to the respirator thickness, about 1 mm. For the NaCl particles (*ρ* = 2250 kg/m^3^, *d*_*p*_ = 100 nm), the Stokes number is on the order of 0.0001. For the influenza droplets (*ρ* = 1000 kg/m^3^, *d*_*p*_ = 5 microns), the Stokes number is on the order of 0.1. Given that the Stokes numbers are significantly less than 1 for the 10 LPM flow rate, we conclude that separation from streamlines is unlikely for both the NaCl particles and influenza droplets. At the 70 LPM flow rate, the Stokes number for the influenza droplets approaches 1, and some separation is presumably possible. We note, however, that as the air flows around the tip of the respirator, separation will occur toward the center of the channel (the approximate gap geometry), not the respirator surface, making adhesion to the channel unlikely. We conclude that the particle leakage rate based upon the 100 nm NaCl particles is a reasonable approximation to the leakage rate for 5-micron influenza droplets. Sample CFD calculations using both 100 nm and 5-micron sizes also showed no difference in particle leakage rate for the two particle sizes. The similar leakage rates lead to similar CIR predictions from the risk-assessment model.

In this study, direct measurements of the porous-media properties of the respirator were not made. In lieu of that, pressure drop measurements were used to calibrate the porous media model to match the experiment results. This approach was used for our study since the focus was to understand the leakage flow around the respirator and not the flow through the porous layers of the mask. Airflow and filtration properties of the porous layers of respirators are well studied and reported elsewhere. Recent studies have shown that the aerosol transmission can be significantly affected by factors such as humidity and fiber involvement in aerosol growth inside the porous layers of the face masks^32,33^.

The leakage gaps for this study were captured by donning the respirators to the rigid 3D printed mannequins. In reality, when the respirators are donned by humans, the respirator will contact the human skin and/or facial hair, which is more compliant than the mannequin material. In addition, during a breathing cycle, air inhalation and exhalation will cyclically change the contact dynamics between the face and the respirator. These complexities were not addressed in the study, as a rigid mannequin and steady suction was used in the experiments. Previous studies have designed respirable mannequins that are capable of mimicking the breathing cycle^34^. The current approach can accommodate such a mannequin provided that the scan frequency of the CT image is higher than the breathing cycle of the mannequin. The CT imaging and reconstruction approach could then capture the dynamic changes in the gap profile during the breathing cycle. Typically, the spatial resolution of a CT scan is inversely proportional to the temporal resolution. Hence, the frequency of the CT scans should be selected so that the spatial resolution is smaller than the size of the smallest gap expected for a headform-respirator combination. The expected aerosol leakage for each acquired gap profile can be computed from the CFD model, and an average (over the breathing cycle) aerosol flux input into the lung deposition model.

## Conclusions

Computational models informed by CT images of gap profiles were employed to predict the risk of infection due to aerosol leakage caused by imperfect fitting of N95 respirators. The CFD model was validated using experiments performed on rigid acrylic mannikins. Depending upon the fit, the leakage values predicted by the CFD simulations varied between 30% and 95%. Calculations of cumulative infection rate showed that a lack of fit testing can increase risk of infection by an order of magnitude, though the risk is substantially less than that for no protection. The set of computational models presented are useful for predicting the relative infection risk for different types of protection, facial profile types, pathogen characteristics, and level of compliance. The leakage and the risk-assessment models can be helpful in developing protection strategy, establishing PPE guidelines, and promoting awareness for the general public during a pandemic.
